# The Effects of Outreach Initiatives on Equitable Access to Dermatology Services: Single-Center Retrospective Analysis

**DOI:** 10.2196/94872

**Published:** 2026-09-11

**Authors:** J Mark Albrecht, Justin David Lyon, Michael Birdsall, Eliza Broadbent, Nicole Ufkes, Stephanie Zone, Anneli Bowen, Christopher Hull, Lauren Madigan, Ashley M Snyder, Chaorong Wu, Bethany K Lewis

**Affiliations:** 1 School of Medicine, University of Utah, Salt Lake City, UT, United States; 2 Department of Dermatology, University of Utah Hospital, 30 Mario Capecchi Drive, HELIX Building, Salt Lake City, UT, 84112, United States, 1 801-581-6465; 3 Department of Population Health, University of North Dakota, Grand Forks, ND, United States; 4 Department of Population Health Sciences, University of Utah, Salt Lake City, UT, United States; 5 Division of Epidemiology, Department of Internal Medicine, University of Utah, Salt Lake City, UT, United States

**Keywords:** disparities, dermatology, outreach, access, rural, underserved

## Abstract

**Background:**

Maldistribution of providers, differences in insurance coverage and acceptance, and underrepresentation of underserved patient populations in dermatology all affect patient access to dermatology services. Few studies have evaluated institutional models to address these pervasive gaps.

**Objective:**

The aim of this study was to evaluate one academic institution’s efforts to comprehensively expand access to dermatology services.

**Methods:**

A retrospective analysis was performed using data from the University of Utah Health (UUH) Department of Dermatology’s outreach initiatives compared with general dermatology ambulatory clinics on UUH’s main campus in Salt Lake City, Utah, over 3 years. Descriptive statistics were reported for patient demographics, insurance coverage, and diagnoses at the encounter level. Statistical methods assessed differences between clinic types. Outreach initiatives were categorized by delivery method: rural in-person clinics, urban in-person charity care clinics, live video teledermatology in rural and incarcerated settings, and asynchronous eConsults (store-and-forward teledermatology).

**Results:**

Outreach initiatives served 2283 distinct patients in 3471 encounters. The general dermatology clinics at UUH served 207,493 patients in 1,141,280 encounters. Overall, UUH outreach initiatives served a more diverse, younger, and Medicaid-insured patient population with a higher prevalence of inflammatory/autoimmune conditions than its general ambulatory dermatology clinics. eConsult and charity care clinics more commonly served patients younger than 65 years of age, and eConsults in particular served a more racially and ethnically diverse patient population compared with the general dermatology clinics.

**Conclusions:**

This study confirms that UUH Dermatology’s efforts to enhance health care access effectively reach targeted underserved patient populations that are underrepresented at UUH’s general dermatology clinics.

## Introduction

Between 1974 and 2000, office visits to dermatologists in the United States approximately doubled, increasing from about 18 million to about 36 million [1]. Density of dermatology providers also increased overall. However, pervasive gaps in dermatology access continue to exist for underserved populations [2]. Underserved populations are defined as those lacking adequate access to health care, including racial and ethnic minority groups, rural populations, persons of lower socioeconomic status, incarcerated populations, children, LGBTQ+ (lesbian, gay, bisexual, transgender, queer) populations, and older adults, among others [3]. While the factors surrounding these gaps are complex, dermatology workforce distribution and insurance acceptance policies are likely substantial contributors.

Challenges in accessing care are likely shaped by the uneven geographic distribution of providers. In this article, providers are defined as board-certified dermatologists and nonphysician midlevel providers. In 2013, urban counties had approximately 50 times the density of dermatology providers as rural counties [4]. Geographic disparities are particularly pronounced within pediatric dermatology, with 96.4% of pediatric dermatologists practicing in urban areas and 3.6% in rural areas [5]. A survey of over 2000 members of the American Academy of Dermatology revealed that nearly half of respondents in rural communities endorsed a need for more dermatologists, while over one-third of urban and suburban dermatologists reported an oversupply in their communities [6]. A higher density of dermatologists has been associated with improved survival of more severe conditions, such as Merkel cell carcinoma [7]. Specialist shortages, by contrast, have been linked to longer visit wait times and greater travel distances, with concomitant negative impacts on treatment adherence and follow-up [8].

Even if provider density is adequate, insurance status affects access to care. Patients with Medicaid are less than half as likely as those with private insurance to receive a skin-related diagnosis from a dermatologist [9]. Creadore et al [10] conducted a secret shopper study of 611 dermatology clinics and found that patients with Medicaid experienced less success in securing an appointment and longer wait times than those with private insurance or Medicare. An analysis of the National Medical Expenditure Panel Survey found that patients with Medicaid or Medicare and patients with no insurance had reduced odds (0.75 and 0.39, respectively) of receiving outpatient dermatology care compared with patients with private coverage [11]. Publicly insured and uninsured populations accordingly make up a disproportionately small proportion of the general dermatologic patient base [12].

Dermatology workforce idiosyncrasies and insurance acceptance policies likely influence who accesses dermatologic services, possibly affecting the outcomes of underserved patients. Individuals from racial and ethnic minority groups are less likely to visit an outpatient dermatologist and bear a disproportionate burden of skin cancer morbidity and mortality [9,13-15]. Rural patients experience greater travel distances, lower rates of follow-up, and higher mortality rates [7]. Public insurance has been associated with reduced visit frequency, lower acceptance rates, and longer wait times [9,16]. Incarcerated populations have been reported to experience prolonged time to treatment for melanoma [17]. In summary, these findings suggest that significant portions of the US population lack access to dermatologic specialist care.

Academic medical institutions can play a key role in addressing these disparities. These institutions may have the administrative, workforce, and intellectual resources to create programs targeting underserved populations while teaching the next generation of dermatology providers the skills to treat these populations. However, the literature on possible models for academic institutions to expand access to underserved populations is limited and lacks a comprehensive approach. A cross-sectional study assessing community outreach initiatives across dermatology residency programs found that 43% of outreach offerings were focused on skin cancer prevention and screening, while charity care clinics comprised the second most common outreach initiative at 32.4% [18]. Clearly, large pockets of underserved populations—for example, racial and ethnic minority patients in whom skin cancer rates are low overall, rural patients who live hours away from a free clinic or skin cancer screening offering, or incarcerated patients who would not qualify for care via a free clinic—would not be reached by these highly circumscribed initiatives. Over the past 2 decades, University of Utah Health (UUH) has engaged in efforts to provide comprehensive and equitable access to dermatology services. This review aims to summarize and analyze our institution’s outreach efforts in the hope of contributing a potential model for academic institutions to address this pressing issue.

The UUH Department of Dermatology has analyzed the demographics, insurance status, and diagnostic data from most of its outreach efforts to assess whether it is reaching the intended populations and to identify the most common dermatologic diagnoses across different venues.

## Methods

### Descriptive Review of UUH Dermatology Outreach Efforts

#### Overview

**Figure 1.**
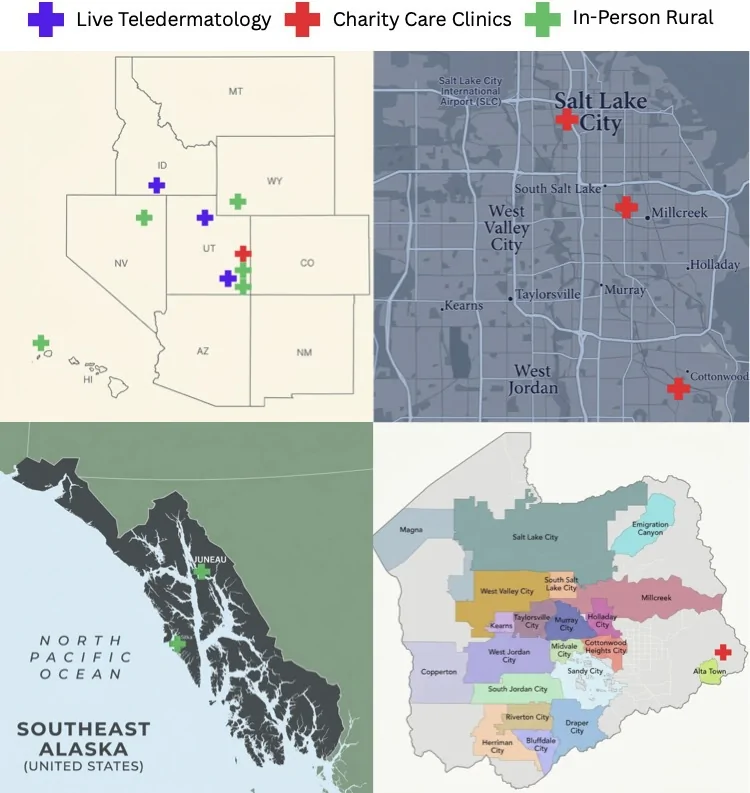
Maps of University of Utah Health (UUH) outreach initiative locations by category. (A) and (C) show locations of outreach initiatives in 6 states. (B) and (D) show outreach initiative locations in the Salt Lake City metropolitan area and adjacent Summit County areas. Map generated using Gemini Flash 3.6.

Outreach requires community collaboration and works to bridge the health care access gaps observed in disadvantaged populations [19]. UUH seeks to increase equitable access to health care through a mixed model of in-person and technology-assisted outreach. UUH is located in Salt Lake County, Utah, which had an immediate surrounding population of 1.19 million people as of 2022 [20,21]. As a uniquely positioned tertiary care center, UUH’s reach extends to a large rural geographic area known as the Intermountain West (Figure 1). Salt Lake County is predominantly White (70.7%), and 8% of its population is enrolled in Medicaid [20,21]. Demographic data indicate that the patient population of UUH’s general dermatology clinics skews White, female, older, and Medicare-insured (unpublished internal data; Table 1) [20,21].

**Table 1.** Comparison of demographic characteristics and insurance status between the populations of Salt Lake County and the University of Utah Health (UUH) general dermatology clinics.

Demographic characteristics	Salt Lake County population (N=1,186,257), n (%)^a^	UUH general dermatology clinic population, n/N (%)^b^
Age (years)		
<18	296,884 (25.0)	61,912/997,869 (6.2)
18‐64	746,293 (62.9)	522,970/997,869 (52.4)
≥65	143,080 (12.1)	412,987/997,869 (41.4)
Sex		
Female	584,135 (49.2)	534,328/997,864 (53.5)
Race^c^		
White	839,228 (70.7)	847,091/870,944 (97.3)
Asian	52,103 (4.4)	13,699/870,944 (1.6)
Pacific Islander	21,429 (1.8)	2,421/870,944 (0.3)
Black	21,707 (1.8)	5,075/870,944 (0.6)
Ethnicity^d^		
Hispanic	233,780 (19.7)	43,399/900,971 (4.8)
Insurance status^e^		
Medicare	109,254 (9.2)	354,326/917,240 (38.6)
Private	705,822 (59.5)	534,774/917,240 (58.3)
Medicaid	96,561 (8.1)	28,140/917,240 (3.1)

a US census data, 2022 [21].

b Denominators differ due to exclusion of missing data for some variables; percentages may not add up to 100 because the “other,” “unknown,” and “self-pay/uninsured” categories are excluded.

c The “unknown” category is excluded from UUH general dermatology clinics data. The “other” category is included in the denominator but not reported here given lack of specificity or direct comparison to county census data.

d The “unknown” category is excluded in UUH general dermatology clinic data.

e The “self-pay/uninsured” category is not included.

#### In-Person Initiatives: Urban Charity Care Clinics and Rural In-Person Clinics

In-person clinics include charity care clinics, which are primarily located in urban centers in Utah, and rural clinics located throughout the Intermountain West and beyond. Textbox 1 provides a concise overview of UUH Dermatology outreach initiatives by type, location, and targeted populations. Charity care clinics are staffed by faculty dermatologists, residents, and/or medical students. Fourth Street Clinic specifically serves persons experiencing homelessness (Salt Lake City, Utah), and Maliheh Clinic (Millcreek, Utah) targets underinsured, uninsured, and Hispanic and Latino patient populations. Midvale Clinic (Midvale, Utah) and People’s Health Clinic (Summit County, Utah) similarly target underinsured, uninsured, and Hispanic patients, and Moab Free Clinic (Moab, Utah) targets underinsured, uninsured, and rural patients; however, data are not yet available from the latter 3 sites. Six rural in-person clinics provide dermatology care to rural populations in the Intermountain West and beyond. Providers fly or drive to these clinics, generally on a monthly basis. Providers spend from 1 day to up to 2 weeks on-site providing care and then manage follow-up care by working either with on-site staff or with UUH Dermatology staff and resources. Currently, clinics take place in Moab, Utah (population of 5317); Rock Springs, Wyoming (population of 23,196); Elko, Nevada (population of 20,613); Montezuma Creek, Utah (population of 324, located in San Juan County, with a population of 18,557); Princeville, Kauai, Hawaii (population of 1918); and Sitka and Juneau, Alaska (populations 8968 and 32,255, respectively). Data from the Montezuma Creek, Kauai, Sitka, and Juneau clinics are not yet available due to restrictions on data access or the new status of these clinics. All site partnerships with both rural in-person and urban charity care clinics arose from the members and staff of the aforementioned clinics, who sought regular dermatologic care from UUH Dermatology; each site has its own unique clinic flow, logistical arrangements, and contractual agreements. Of note, no interstate licensure exists, so multiple state licensures for providers must be applied for and maintained.

Textbox 1. **University of Utah Health (UUH) Dermatology outreach initiatives by type, location, and targeted populations.**

Urban charity care clinics in Utah:

- Fourth Street Clinic, Salt Lake City: persons experiencing homelessness
- Maliheh Clinic, Millcreek: urban under- and uninsured persons and persons of Hispanic ethnicity
- Midvale Clinic, Midvale: urban under- and uninsured persons and persons of Hispanic ethnicity
- People’s Health Clinic, Park City: under- and uninsured persons and persons of Hispanic ethnicity
- Moab Free Clinic, Moab: rural population, under- and uninsured persons

Rural in-person clinics:

- Elko, Nevada: rural population
- Rock Springs, Wyoming: rural population
- Moab, Utah: rural population
- Kauai, Hawaii: rural population
- Montezuma Creek, Utah: rural population, Native American persons
- Sitka and Juneau, Alaska: rural population, Native Alaskan persons

Live video teledermatology:

- Salt Lake County Jail, Utah: incarcerated population
- Utah Prison, Utah: incarcerated population
- Blanding, Utah: rural population
- Gooding, Idaho: rural population

Store-and-forward eConsults:

- Medicaid users, children, racial and ethnic minority groups

#### Telehealth Initiatives: Live Video Teledermatology and Store-and-Forward eConsults

Teledermatology provides several unique methods of outreach, with studies indicating similar clinical outcomes as in-person dermatology services [22,23]. The use of teledermatology (synchronous live video and asynchronous eConsults) in the UUH Dermatology outreach program has expanded the breadth of coverage to both rural and incarcerated settings. For rural populations, live video teledermatology appointments are offered in Blanding, Utah (population of 3319), and Gooding, Idaho (population of 3713). Similar programs have been established at the Utah Prison (occurring monthly since 2003) and Salt Lake County Jail (occurring weekly since 2020). It should be emphasized that incarcerated persons would have severely limited access to dermatologic specialty services without live video teledermatology. Additionally, store-and-forward teledermatology has been offered under the UUH umbrella by means of our eConsult service since 2019; notably, eConsults are covered by Medicaid in Utah. UUH providers submit a history and photographs of the dermatologic issue of interest, which is reviewed by a faculty dermatologist who provides recommendations. This method has been shown by other institutions to decrease patient appointment wait times and provide cost savings to patients and health care systems without sacrificing diagnostic accuracy or patient satisfaction [24].

### Study Design

Data from UUH outreach initiatives and general dermatology clinics at the encounter level over a 3-year period were extracted and compared. Differences between outreach initiatives and general dermatology clinics were examined in terms of demographics, insurance status, and diagnostic categories. Diagnostic codes from patient encounters were categorized and classified as benign growth, malignant lesion, premalignant lesion, infectious process, inflammatory/autoimmune process, wound/burn, or other. Total numbers and percentages were reported at the encounter level for insurance, gender, ethnicity, race, age group, and diagnostic category. *t* tests were used to assess continuous variables. *χ*² tests or Fisher exact tests were used, as appropriate, to investigate categorical variables. Outreach initiatives were further classified and analyzed based on delivery method, distinguishing between (1) rural in-person clinics (Elko, Moab, and Rock Springs), (2) live video teledermatology appointments (subcategorized as rural [Gooding and Blanding] and incarcerated [Salt Lake County Jail and Utah Prison]), (3) charity care clinics (Maliheh Clinic and Fourth Street Clinic), and (4) eConsults. All analyses were performed with R software (version 4.3.1; R Foundation for Statistical Computing).

### Ethical Considerations

This retrospective chart review was reviewed by the University of Utah Institutional Review Board and determined to be exempt from institutional review board approval (IRB #76927). The study was conducted in accordance with applicable institutional and ethical standards for research involving human subjects.

As this study involved a retrospective review of existing medical records, informed consent was not obtained from participants. Patient privacy and confidentiality were maintained throughout the study. Data were deidentified prior to analysis, and no names, medical record numbers, or other directly identifying information were included in the study dataset or manuscript. Study data were securely stored and accessible only to authorized members of the research team. No participant compensation was provided.

Figure 1 was generated using a generative AI image model, Gemini (Flash 3.6; Google), accessed through the Health Insurance Portability and Accountability Act (HIPAA)–secure University of Utah Workspace environment. The image was produced through an iterative prompting process: an initial prompt requested a generic map of (map/topic), drawing on user-supplied reference examples and publicly available online sources. Subsequent prompts were used to refine the output (eg, narrowing the geographic scope, removing highway labels, and adjusting background color) until the final version met the intended presentation requirements. No patient data, protected health information, or identifiable images were used in generating this figure. The AI-generated image was reviewed by the authors for accuracy prior to inclusion in the manuscript.

## Results

**Figure 2.**
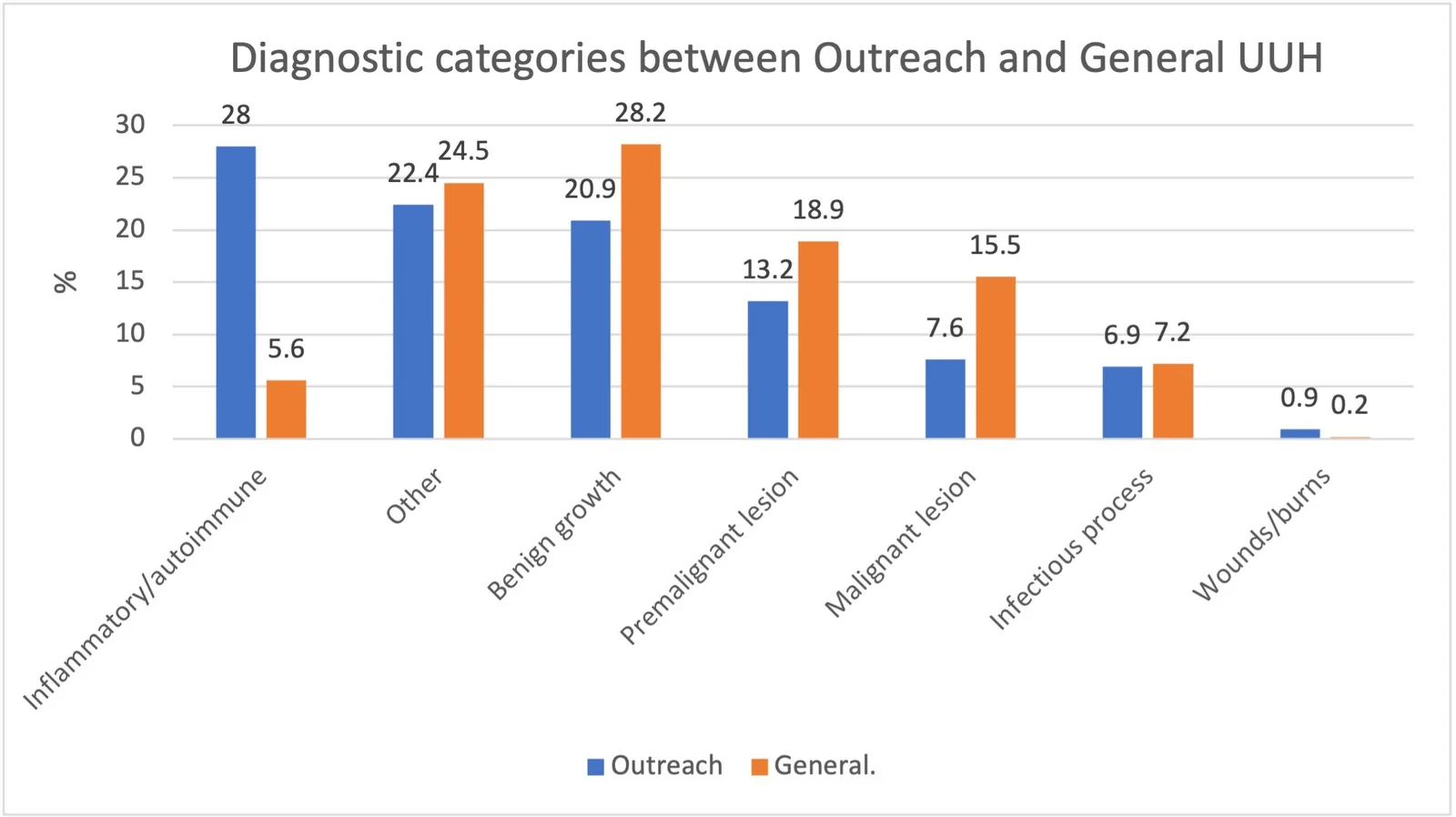
Percentage of diagnoses by diagnostic category among University of Utah Health (UUH) outreach initiative methods and general dermatology clinics.

In total, 3471 encounters (2283 distinct patients) occurred across all UUH outreach initiatives, while the university’s general dermatology clinics reported 1,141,280 encounters (207,493 patients). A comparison of demographics and insurance coverage between outreach settings and general dermatology clinics at the encounter level is reported in Table 2. Results are significant (*P*<.001) unless otherwise noted. Overall, encounters in outreach settings were more commonly associated with patients covered by Medicaid than encounters at UUH general dermatology clinics (n=205, 8.3% vs n=28,140, 2.5%). Gender differences were also observed, with male patients seen in significantly more encounters at outreach settings than in general dermatology clinics (n=1886, 54.3% vs n=463,536, 46.5%). Furthermore, compared with general dermatology clinics, significantly more encounters in outreach settings were with patients of Hispanic/Latino (n=309, 11.1% vs n=43,399, 3.8%), American Indian (n=156, 4.7% vs n=2658, 0.3%), Black/African American (n=74, 2.2% vs n=5075, 0.5%), or Native Hawaiian or Pacific Islander race or ethnicity (n=31, 0.9% vs n=2421, 0.2%). Consequently, an overall lower proportion of encounters in outreach settings were with White/Caucasian patients (n=2570, 77.5%) compared with general dermatology clinics (n=847,091, 85.1%). The mean age of patients in encounters across all outreach initiatives was younger than in general dermatology clinics (49.4, SD 18.8 years vs 55.5, SD 20.9 years), with a greater percentage falling in the age range of 18 to 64 years (n=2041, 71.3% vs n=522,970, 52.4%). Diagnostically, a higher proportion of inflammatory/autoimmune diseases (n=1169, 28.0% vs n=60,951, 5.6%) and a lower proportion of malignant (n=318, 7.6% vs n=168,136, 15.5%), premalignant (n=551, 13.0% vs n=204,657, 18.9%), and benign growths (n=871, 20.5% vs n=305,619, 28.2%) were seen across all outreach settings versus general dermatology clinics (Figure 2).

**Table 2.** Demographics and insurance comparison between patient encounters in University of Utah Health (UUH) outreach and general clinic settings.

	Outreach encounters (n=3471)	UUH general clinic encounters (n=1,141,280)	Total encounters (N=1,144,751)	*P* value
Age (years), mean (SD)^a^	49.4 (18.8)	55.5 (20.9)	55.4 (20.8)	<.001
Age (years), n (%)^a^				<.001
<18	191 (6.7)	61,912 (6.2)	62,103 (6.2)	
18‐64	2041 (71.3)	522,970 (52.4)	525,011 (52.5)	
≥65	631 (22.0)	412,987 (41.4)	413,618 (41.3)	
Insurance, n (%)^b^				<.001
Medicaid	205 (8.3)	28,140 (2.5)	28,345 (2.5)	
Medicare	771 (31.3)	354,326 (31.0)	355,097 (31.0)	
Private	1168 (47.5)	534,774 (46.9)	535,942 (46.9)	
Self-pay/uninsured	316 (12.8)	224,040 (19.6)	224,356 (19.6)	
Gender, n (%)^c^				<.001
Female	1585 (45.7)	534,328 (53.5)	535,913 (53.5)	
Male	1886 (54.3)	463,536 (46.5)	465,422 (46.5)	
Ethnicity, n (%)^d^				<.001
Hispanic/Latino	309 (11.1)	43,399 (3.8)	43,708 (3.8)	
Not Hispanic/Latino	2051 (73.8)	857,572 (75.1)	859,623 (75.1)	
Unknown/declined to answer	419 (15.1)	240,309 (21.1)	240,728 (21.0)	
Race, n (%)^e^				<.001
American Indian or Alaskan Native	156 (4.7)	2658 (0.3)	2814 (0.3)	
Asian	25 (0.8)	13,699 (1.4)	13,724 (1.4)	
Black/African American	74 (2.2)	5075 (0.5)	5149 (0.5)	
Native Hawaiian or other Pacific Islander	31 (0.9)	2421 (0.2)	2452 (0.2)	
Other	128 (3.9)	38,073 (3.8)	38,201 (3.8)	
Unknown	330 (10.0)	86,128 (8.7)	86,458 (8.7)	
White/Caucasian	2570 (77.5)	847,091 (85.1)	849,661 (85.1)	

a Missing: n=1011.

b Missing: n=143,416.

c Missing: n=692.

d Missing: n=146,292.

e Missing: n=144,019.

Distinctions in demographics and insurance coverage between methods of outreach (teledermatology vs in person) to similar rural locations and general dermatology clinics were also observed (Table 3). Findings are significant (*P*<.001) unless otherwise noted. Medicaid use was proportionally higher in live video teledermatology encounters (Blanding: 34/150, 25.0%; Gooding: 24/103, 27.0%) compared with general dermatology clinics (28,140/1,141,280, 2.5%). Furthermore, live video teledermatology encounters were more commonly with American Indian and Alaska Native patients (Blanding: 69/158, 43.7%; Gooding: 45/103, 43.7%) compared with general dermatology clinics (2658/1,141,280, 0.3%). Conversely, significantly higher proportions of rural in-person clinic encounters involved non-Hispanic patients (Moab: 641/762, 84.1%; Rock Springs: 460/528, 87.1%) compared with general dermatology clinics (857,572/1,141,280, 75.1%). Notably, patients younger than 18 years of age were more commonly seen in encounters via live video teledermatology (Blanding: 23/158, 14.6%; Gooding: 20/103, 19.4%) than rural in-person clinics (Elko: 44/427, 10.3%; Moab: 36/762, 9.1%; Rock Springs: 21/528, 7.3%) or general dermatology clinics (61,912/1,141,280, 6.2%).

**Figure 3.**
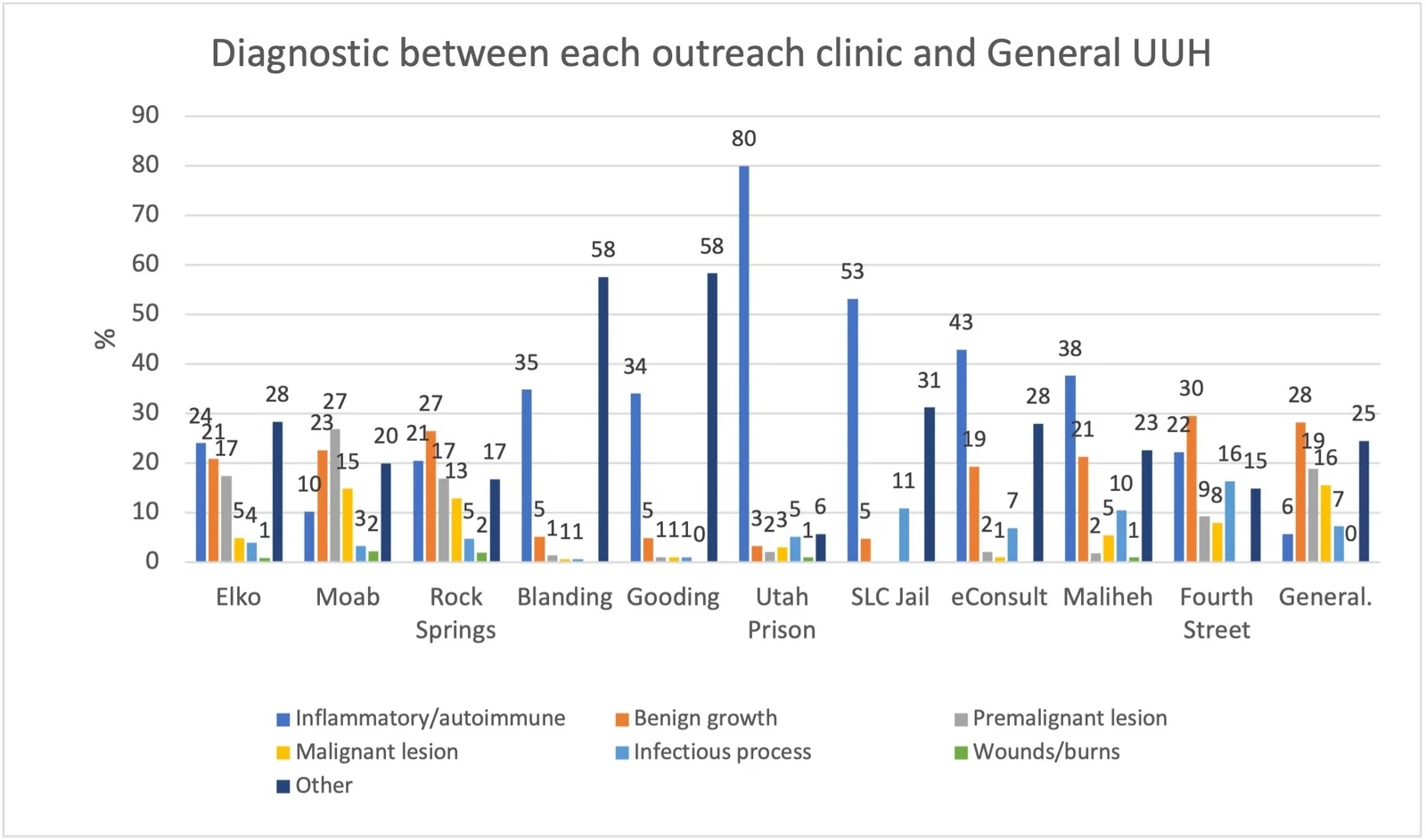
Prevalence of diagnostic categories at individual outreach initiative locations and general dermatology clinics. UUH: University of Utah Health; SLC: Salt Lake County.

Regarding diagnostic categories at the encounter level, rural in-person clinics more commonly saw a higher proportion of benign growths (Elko: 193/926, 20.8%; Moab: 172/263, 22.6%; Rock Springs: 140/528, 26.5%) compared with live video teledermatology services to rural locations (Blanding: 8/158, 5.1%; Gooding: 5/103, 4.9%), while live video teledermatology services to rural locations diagnosed more inflammatory/autoimmune processes (Blanding: 55/158, 34.8%; Gooding: 35/103, 34.0%) compared with general dermatology clinics (60,951/1,408,744, 5.6%). Findings are summarized in Figure 3 and expanded upon in Table 4.

**Table 3.** Demographics and insurance coverage of patient encounters at each outreach initiative location and the University of Utah Health (UUH) general clinics.

	Elko (n=427)	Moab (n=762)	Rock Springs (n=528)	Blanding (n=158)	Gooding (n=103)	Utah Prison (n=336)	SLC^a^ Jail (n=64)	eConsult (n=233)	Maliheh (n=234)	Fourth Street (n=626)	General UUH (n=1,141,280)	*P* value
Insurance, n (%)^b^												<.001
Medicaid	63 (15.2)	19 (2.5)	12 (2.3)	34 (25.0)	24 (27.0)	—^c^	1 (1.6)	52 (22.3)	—	—	28,140 (2.5)	
Medicare	126 (30.4)	347 (45.5)	184 (34.8)	49 (36.0)	29 (32.6)	—	4 (6.2)	32 (13.7)	—	—	354,326 (31.0)	
Private	225 (54.3)	352 (46.2)	305 (57.8)	53 (39.0)	36 (40.4)	—	59 (92.2)	138 (59.2)	—	—	534,774 (46.9)	
Self-pay/uninsured	0 (0)	44 (5.8)	27 (5.1)	0 (0)	0 (0)	—	—	11 (4.7)	234 (100.0)	—	224,040 (19.6)	
Gender, n (%)^d^												<.001
Female	264 (61.8)	415 (54.5)	304 (57.6)	68 (43.0)	45 (43.7)	32 (9.5)	2 (3.1)	114 (48.9)	148 (63.2)	193 (30.8)	534,328 (53.5)	
Male	163 (38.2)	347 (45.5)	224 (42.4)	90 (57.0)	58 (56.3)	304 (90.5)	62 (96.9)	119 (51.1)	86 (36.8)	433 (69.2)	463,536 (46.5)	
Ethnicity, n (%)^e^												<.001
Hispanic/Latino	40 (9.4)	10 (1.3)	27 (5.1)	3 (1.9)	2 (1.9)	43 (12.8)	9 (14.1)	40 (17.2)	135 (57.7)	—	43,399 (3.8)	
Not Hispanic/Latino	301 (70.5)	641 (84.1)	460 (87.1)	127 (80.4)	82 (79.6)	186 (55.4)	37 (57.8)	185 (79.4)	32 (19.0)	—	857,572 (75.1)	
Unknown/declined	86 (20.1)	111 (14.6)	41 (7.8)	28 (17.7)	19 (18.4)	107 (31.8)	18 (28.1)	1 (0.6)	0 (0)	—	240,309 (21.1)	
Race, n (%)^f^												<.001
American Indian or Alaskan Native	17 (4.0)	2 (0.3)	2 (0.4)	69 (43.7)	45 (43.7)	1 (0.4)	—	2 (0.9)	1 (0.7)	17 (2.7)	2658 (0.3)	
Black/African American	0 (0)	3 (0.4)	0 (0)	1 (0.6)	1 (1.0)	6 (2.3)	5 (7.8)	7 (3.0)	9 (6.0)	42 (6.7)	5075 (0.5)	
Native Hawaiian or other Pacific Islander	2 (0.5)	0 (0)	0 (0)	4 (2.5)	4 (3.9)	1 (0.4)	10 (15.6)	6 (2.6)	2 (1.3)	2 (0.3)	2421 (0.2)	
Other	5 (1.2)	8 (1.0)	19 (3.6)	6 (3.8)	4 (3.9)	42 (16.0)	9 (14.1)	35 (15.0)	—	—	38,073 (3.8)	
Unknown	76 (17.8)	109 (14.3)	37 (7.0)	27 (17.1)	18 (17.5)	30 (11.5)	18 (28.1)	6 (2.6)	1 (0.7)	8 (1.3)	86,128 (8.7)	
White/Caucasian	327 (76.6)	639 (83.9)	467 (88.4)	51 (32.3)	31 (30.1)	178 (67.9)	22 (34.4)	165 (70.8)	138 (91.4)	552 (88.2)	847,091 (85.1)	
Age (years), mean (SD)^g^	51.1 (21.4)	54.5 (20.5)	56.5 (20.1)	54.7 (23.6)	51.7 (24.6)	41.0 (13.1)	44.3 (11.6)	38.4 (20.9)	48.7 (16.7)	49.5 (10.4)	55.5 (20.9)	<.001
Age (years), n (%)^g^												<.001
<18	44 (10.3)	36 (9.1)	21 (7.3)	23 (14.6)	20 (19.4)	0 (0.0)	0 (0.0)	37 (15.9)	10 (4.3)	0 (0.0)	61,912 (6.2)	
18‐64	244 (57.1)	196 (49.4)	137 (47.9)	79 (50.0)	50 (48.5)	318 (94.9)	62 (96.9)	170 (73.0)	183 (78.2)	602 (96.2)	522,970 (52.4)	
≥65	139 (32.6)	165 (41.6)	128 (44.8)	56 (35.4)	33 (32.0)	17 (5.1)	2 (3.1)	26 (11.2)	41 (17.5)	24 (3.8)	412,987 (41.4)	

a SLC: Salt Lake County.

b Missing: n=1011.

c Not applicable.

d Missing: n=143,416.

e Missing: n=692.

f Missing: n=146,292.

g Missing: n=144,019.

**Table 4.** Diagnoses by category at individual outreach initiatives and University of Utah Health (UUH) general clinics (N=1,089,488; missing, n=323,495).

	Elko	Moab	Rock Springs	Blanding	Gooding	Utah Prison	SLC^a^ Jail	eConsult	Maliheh	Fourth Street	UUH general clinics	*P* value
Total diagnoses, n (%)	926 (0.07)	762 (0.05)	528 (0.04)	158 (0.01)	103 (0.01)	336 (0.02)	64 (0.00)	233 (0.02)	291 (0.02)	838 (0.06)	1,408,744 (99.70)	—^b^
Diagnostic category, n (%)												<.001
Benign growth	193 (20.8)	172 (22.6)	140 (26.5)	8 (5.1)	5 (4.9)	11 (3.3)	3 (4.7)	45 (19.3)	47 (21.3)	247 (29.5)	305,619 (28.2)	
Infectious process	36 (3.9)	25 (3.3)	25 (4.7)	1 (0.6)	1 (1.0)	17 (5.1)	7 (10.9)	16 (6.9)	23 (10.4)	137 (16.3)	78,607 (7.2)	
Inflammatory/ autoimmune process	222 (24.0)	78 (10.2)	108 (20.5)	55 (34.8)	35 (34.0)	268 (80.0)	34 (53.1)	100 (42.9)	83 (37.6)	186 (22.2)	60,951 (5.6)	
Malignant lesion	45 (4.9)	113 (14.8)	68 (12.9)	1 (0.6)	1 (1.0)	10 (3.0)	0 (0)	2 (0.9)	12 (5.4)	66 (7.9)	168,136 (15.5)	
Premalignant lesion	161 (17.4)	205 (26.9)	89 (16.9)	2 (1.3)	1 (1.0)	7 (2.1)	0 (0)	5 (2.1)	4 (1.8)	77 (9.2)	204,657 (18.9)	
Wounds/burns	7 (0.8)	17 (2.2)	10 (1.9)	0 (0)	0 (0)	3 (0.9)	0 (0)	0 (0)	2 (0.9)	0 (0)	1773 (0.2)	
Other	262 (28.3)	152 (19.9)	88 (16.7)	91 (57.6)	60 (58.3)	19 (5.7)	20 (31.2)	65 (27.9)	50 (22.6)	125 (14.9)	265,577 (24.5)	

a SLC: Salt Lake County.

b Not applicable.

Encounters via live video teledermatology for incarcerated populations (Utah Prison and Salt Lake County Jail) and eConsults were also compared with UUH general dermatology in terms of demographics and insurance status (Table 3) and diagnostic data (Figure 3, Table 4). Results are significant (*P*<.001) unless otherwise noted. A significant predominance of encounters in incarcerated settings were with male patients (Utah Prison: 304/336, 90.5%; Salt Lake County Jail: 62/64, 96.9%) and patients between the ages of 18 and 64 years (Utah Prison: 318/336, 94.9%; Salt Lake County Jail: 62/64, 96.9%) compared with general dermatology clinics (463,536/1,141,280, 46.5%, and 522,970/997,869, 52.4%, respectively). In incarcerated settings, diagnostic categorization revealed a significantly higher proportion of inflammatory/autoimmune processes (Utah Prison: 268/336, 80%; Salt Lake County Jail: 34/64, 53.1%) compared with general dermatology clinics (60,951/1,408,744, 5.6%). Encounters via asynchronous eConsult services were more commonly with patients covered by Medicaid (52/233, 22.3%) compared with general dermatology clinics (28,140/1,141,280, 2.5%), and a larger proportion of eConsult encounters were with Hispanic/Latino patients (40/233, 17.2% vs 43,399/1,141,280, 3.8%). Of note, 100 of 233 (42.9%) diagnoses made via eConsult were of inflammatory/autoimmune processes, a significantly higher proportion than at general dermatology clinics.

Finally, an analysis of demographics and insurance coverage (Table 3) and diagnostic data (Figure 3, Table 4) from charity care clinics revealed that encounters at Maliheh Clinic were more commonly with Hispanic/Latino patients (135/234, 57.7%) compared with general dermatology clinics (43,399/1,141,280, 3.8%). A higher percentage of encounters at Fourth Street Clinic compared with general dermatology clinics were with American Indian or Alaskan Native patients (17/626, 2.7% vs 2658/1,141,280, 0.3%) and Black/African American patients (42/626, 6.7% vs 5075/1,141,280, 0.5%); findings are statistically significant (*P*<.001) unless otherwise noted. Encounters at both charity care clinics were disproportionately with patients 18 to 64 years of age (Maliheh: 183/234, 78.2%; Fourth Street Clinic: 602/626, 96.2%) compared with general dermatology clinics (522,970/1,141,280, 52.4%). Further, encounters at the charity care clinics were with a greater proportion of patients with inflammatory/autoimmune conditions (Maliheh: 83/291, 37.6%; Fourth Street Clinic: 186/838, 22.2%) and infectious processes (Maliheh: 23/291, 10.4%; Fourth Street Clinic: 137/838, 16.3%) compared with general dermatology clinics (60,951/1,408,744, 5.6%, and 78,607/1,408,744, 7.2%, respectively).

## Discussion

### Principal Findings

This retrospective analysis demonstrates that the UUH Department of Dermatology’s outreach initiatives serve a meaningfully distinct patient population from that of the institution’s general dermatology clinics. Outreach patients were younger (mean age 49.4, SD 18.8 years vs 55.5, SD 20.9 years), more racially and ethnically diverse, and more likely to be Medicaid-insured, differences that reflect the deliberate design of our outreach locations and partnerships. However, when interpreting these findings, we must acknowledge that the data are skewed by the predominantly White, non-Hispanic, female, older than 65 years, and Medicare-insured population served by our main academic medical campus. This problem is not unique to Utah. Access inequities in dermatology are well documented throughout the United States and are likely amplified by provider distribution and insurance acceptance policies within the field of dermatology. Urban areas commonly have a higher concentration of dermatologists, creating care deserts in rural communities. While the literature has thoroughly characterized these disparities, practical models for academic medical institutions to systematically address them remain scarce.

UUH Dermatology has pursued a multifaceted strategy to fill this gap, deliberately extending care to rural populations, racial and ethnic minority groups, and populations who are uninsured, Medicaid-insured, or incarcerated. These groups are of particular interest given that they are rarely captured in national dermatology use data and often experience the steepest barriers to access [9]. The higher proportion of Hispanic/Latino patients across outreach encounters compared with general dermatology clinics (309/3471, 11.1% vs 43,708/1,144,751, 3.8%; *P*<.001) reflects the intentional design of specific outreach initiatives rather than aggregate coincidence. Maliheh Clinic serves under- and uninsured persons (234/234, 100%) and Hispanic/Latino populations (135/234, 57.7%). Aggregate outreach statistics, therefore, should not be interpreted as uniform across sites. Rather, the targeted nature of individual clinics must be considered when drawing conclusions from pooled data.

This interpretive nuance extends in the other direction as well. Given that the rural Intermountain West skews White, non-Hispanic, older, and Medicare-insured and that rural outreach encounters outnumber charity care clinic encounters in absolute terms, the degree of demographic diversification achieved across outreach sites is even more notable than the raw data may initially suggest. The regional population is predominantly rural and includes a high proportion of Medicare users; the outreach program’s success in reaching people outside of these groups makes the findings more noteworthy, not less.

The diagnostic distribution across outreach settings likewise reflects structural realities. The markedly higher proportion of inflammatory and autoimmune diagnoses (28.0% vs 5.6%) made through outreach initiatives is unsurprising for several reasons. Teledermatology, which comprises a substantial portion of outreach activity, is less suited to lesion-of-concern evaluations and opportunistic skin checks, which often correlate with neoplastic diagnoses in traditional clinic settings. Additionally, more racially and ethnically diverse patient populations carry a lower baseline burden of skin cancer and are more likely to present with inflammatory dermatoses. This pattern is prevalent at the site level: the highest rates of inflammatory and autoimmune diagnoses were found at the Utah State Prison (268/336, 80.0%) and Salt Lake County Jail (34/64, 53.1%), both of which serve predominantly male patients aged 18 to 64 years. Similarly, inflammatory and autoimmune conditions accounted for 100 of 233 (42.9%) diagnoses made through the eConsult service, consistent with the younger, Medicaid-insured, and racially and ethnically diverse patient population it serves. These patterns have direct implications for clinical planning, informing provider expertise, formulary access, and downstream referral resources tailored to each outreach site.

With a model that uses a variety of outreach methods, including rural in-person clinics, urban charity care clinics, live video teledermatology for rural and incarcerated populations, and store-and-forward eConsults, the UUH Department of Dermatology delivers care to pockets of the population that lack access to more traditional clinics. Future goals include expanding this analysis to encompass all current outreach services, including clinics in Montezuma Creek, Utah; Kauai, Hawaii; and Sitka and Juneau, Alaska, after data transfer agreements have been established. Beyond descriptive analyses, future studies may address clinical outcomes and patient-reported experiences, evaluate the cost-effectiveness of each delivery modality, and determine if sustained outreach contributes to reducing delayed diagnosis rates for dermatologic conditions in underserved communities. Longitudinal data would also shed light on continuity of care and adherence to follow-up recommendations, metrics that are essential for understanding whether intentional outreach translates to improved health equity in a concrete and measurable manner.

### Limitations

This study carries several limitations. As a single-center, retrospective, descriptive analysis, these findings reflect the particular geography, infrastructure, and patient population of the Intermountain West. This region largely comprises a White, non-Hispanic, older, Medicare-insured population, which shapes outreach demographics in ways that may not generalize to institutions in more urban or racially diverse settings.

Furthermore, data from newer outreach partnerships and sites without active data transfer agreements were not included in this study, limiting the comprehensiveness of the current analysis. Additionally, eConsult services are currently reimbursed by Medicaid in Utah, which facilitates access for publicly insured patients in this state. However, Medicaid coverage of eConsults varies widely by state, which may limit the direct applicability of this model to institutions in states without such reimbursement privileges.

Race and ethnicity data were self-reported and collected using varied instruments across outreach locations, which may limit direct comparison between sites. Race and ethnicity were recorded as separate variables; patients identifying as Hispanic under ethnicity may also have identified as White/Caucasian under race, which could have contributed to the apparent overrepresentation of White patients in aggregate data.

This analysis captures demographic, insurance coverage, and diagnostic data at the encounter level without long-term clinical outcomes, patient-reported satisfaction, or measures of downstream health care use. These factors would be necessary to complete a comprehensive overview of the contributions these outreach initiatives make to health equity. Finally, given the retrospective, descriptive nature of this study, the analysis is designed to characterize outreach populations rather than to establish causal relationships or evaluate the efficacy of specific outreach modalities. The aim of future work is to provide a comprehensive evaluation to allow for the fullest and most accurate picture of the populations served, diagnoses seen, and impact on reducing access barriers in underserved populations.

### Conclusions

This analysis of the UUH Department of Dermatology’s clinical outreach services demonstrates that these initiatives reach distinct underserved patient populations compared with those seen in the institution’s general dermatology ambulatory clinics. The breadth and deliberate design of these initiatives offer a working model for how academic medical institutions can move beyond describing health disparities and toward actively addressing them.

Academic dermatology departments are particularly well positioned to lead this work because they can forge community partnerships and have the administrative infrastructure and workforce depth necessary to operate across multiple delivery modalities simultaneously. We present this model and its evaluation as a starting point for cross-institutional dialogue about how dermatology providers can more equitably reach the patients who need these services the most.

## Abbreviations

HIPAA: Health Insurance Portability and Accountability Act
LGBTQ+: lesbian, gay, bisexual, transgender, queer
SLC: Salt Lake County
UUH: University of Utah Health
